# T_1_-MRI Fluorescent Iron Oxide Nanoparticles by Microwave Assisted Synthesis

**DOI:** 10.3390/nano5041880

**Published:** 2015-11-04

**Authors:** Riju Bhavesh, Ana V. Lechuga-Vieco, Jesús Ruiz-Cabello, Fernando Herranz

**Affiliations:** 1 Spanish Cardiovascular Research Centre (CNIC) and Spanish Pulmonary Diseases Network (CIBERES) C/Melchor Fernández-Almagro 3, 28029 Madrid, Spain; E-Mails: riju.bhavesh@cnic.es (R.B.); anavictoria.lechuga@cnic.es (A.V.L.-V.); ruizcabe@cnic.es (J.R.-C.); 2 Department of Physicochemistry II, Complutense University of Madrid, 28040 Madrid, Spain

**Keywords:** iron oxide nanoparticles, MRI, T_1_ contrast, microwave

## Abstract

Iron oxide nanoparticles have long been studied as a T_2_ contrast agent in MRI due to their superparamagnetic behavior. T_1_-based positive contrast, being much more favorable for clinical application due to brighter and more accurate signaling is, however, still limited to gadolinium- or manganese-based imaging tools. Though being the only available commercial positive-contrast agents, they lack an efficient argument when it comes to biological toxicity and their circulatory half-life in blood. The need arises to design a biocompatible contrast agent with a scope for easy surface functionalization for long circulation in blood and/or targeted imaging. We hereby propose an extremely fast microwave synthesis for fluorescein-labeled extremely-small iron oxide nanoparticles (fdIONP), in a single step, as a viable tool for cell labeling and T_1_-MRI. We demonstrate the capabilities of such an approach through high-quality magnetic resonance angiographic images of mice.

## 1. Introduction

Iron based nanoparticles have enticed researchers for long, as a potent tool for contrast enhancement in magnetic resonance imaging (MRI) [1,2]. A typical contrast agent is supposed to shorten the transverse, T_2_/T_2_* (negative contrast), or the longitudinal, T_1_ (positive contrast) relaxation times of protons in the water molecules. Iron oxide nanoparticles, for instance, have been widely studied for the former [3,4,5]. T_2_/T_2_* shortening, accounting for the negative contrast, induces the signal from both the target and the surrounding area. Superparamagnetic iron oxide nanoparticles (SPIONs) have, therefore, evolved as optimal negative contrast agents due to their high magnetic moment and high metal payload. This magnetic behavior is translated in very large values for *r*_2_, the transverse relaxivity, making it possible to get large signals in the image using small amounts of contrast materials. However, being their most remarkable property it can also be a burden for many applications in imaging. This is due to the fact that T_2_ pulse sequences are difficult to use for the diagnosis of many pathologies due to the possibility of endogenous negative contrast, which may be produced by calcium depositions, bleeding, or the presence of other metals. This situation is particularly complicated in cardiovascular imaging, for example, in atherosclerotic plaque characterization or angiography. All these factors have contributed to a limited clinical application, even at the expense of using the highly-toxic gadolinium. This has led to a recent and intense research for the production of iron oxide nanoparticles for T_1_ MRI [6,7,8]. Even with these efforts their use for T_1_ contrast is still, to some extent, not extended. For now, Gd- and Mn-based contrast agents stand as the current pioneers of the field, but with many drawbacks [9,10,11]. Positive contrast with iron oxide nanoparticles, giving a high signal to noise ratio and a bright intensity, is of course, clinically, much more favorable, particularly at field strengths smaller than 3 T, which normally is the case. The efforts, therefore, have been in designing probes competent with the Gd-based positive contrast agents, but with an edge over them with the scope for easy surface functionalization, enhanced biocompatibility, and half-life in blood [12]. One of the primal necessities, when considering iron based contrast agents for such a purpose, is the size confinement. Decreasing the size decreases the net magnetic moment of the particles and increases the surface area, accounting further to an increased density of lone pairs of electrons in the valence shells of the iron oxide nanoparticles in the solution. With this principle as the baseline, our aim was to design a small-sized nanoparticulate system.

Microwave synthesis (MWS) has long been used in the field of chemistry for organic synthesis and catalyzed reactions [13,14,15]. However, only recently has this technology been exploited in the field of nanoparticle synthesis. One of the prominent features responsible for this choice was the fast and selective heating [15,16]. The superior performance of MWS is due to dielectric heating: the rapid heating of the sample as the molecular dipoles try to align with the alternating electric field, with more polar solvents and reagents being more efficiently heated. Several reports have described microwave synthesis of Fe_2_O_3_ and Fe_3_O_4_ nanoparticles; however, the reports showing the MWS of iron oxide for positive contrast MRI are scarce [17,18,19,20,21]. The heating efficiency thus, greatly depends on the material under consideration. For instance, fluctuation in the molecular dipoles as an attempt to align with the alternating electric field leads to the temperature modification at the molecular level, giving an edge to the polar solvents as compared to the conventional heating (water bath, oil bath, *etc.*) which is a bulk phenomenon. This technique, hence, is much faster and eliminates any side reactions. Our work here analyzes the various parameters during the fabrication process, governing, hence, the properties of the synthesized nanoparticles and as a result, modulating their varying efficacy as an MR contrast tool. With fluorescein isothiocyanate carboxymethyl dextran as the surface passivating agent, we intended to render *in vivo* biocompatibility and long circulating times for blood-pool applications. We hereby demonstrate the use of these nanoparticles for high-quality positive contrast in MRI of main arteries and small vessels, while achieving these results through an easy one-step protocol.

## 2. Results and Discussion

### 2.1. Microwave Synthesis and Characterization of Fluorescein Isothiocyanate Carboxymethyl Dextran Iron Oxide Nanoparticles

FITC-CM Dextran (4 KDa) coated iron oxide nanoparticles, fdIONP, were synthesized with microwave irradiation as the heat source. The main goal was to produce extremely small nanoparticles so they could produce positive contrast and long circulating times in blood, with the carboxymethyl derivative of this FITC labeled dextran giving an anchor further, for biomodification and fluorescence signal. Dextran was chosen as surface stabilizer due to its biocompatibility, easy *in vivo* degradation, and for its very property of imparting hydrophilicity and, hence, the stealth from the immune system, when considered under circulation. The reaction progressed by hydrazine mediated reduction of the iron (III) chloride hexahydrate salt under continuous stirring and MW heating. The reaction progressed at 100 °C with a ramping time (from room temperature to the set temperature, 100 °C) of 54 s at 240 W of power. This fast ramping time eliminates, to a great extent, any unspecific reactions and increases the homogeneity of the sample. The sample was cooled under 2 min after the completion of the experiment so as to avoid overheating or any variation in the originally defined parameters for the experiment. The purification of the sample was performed by gel filtration chromatography to remove unreacted iron salt and the excess of hydrazine from the sample, while obtaining the pure fdIONP in a preferred solvent.

**Figure 1:**
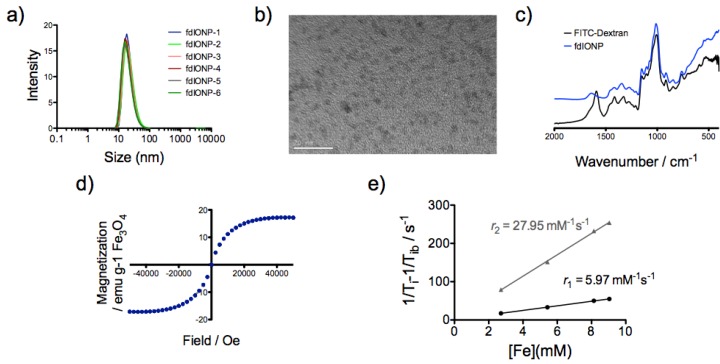
Physicochemical characterization of fdIONP. (**a**) Hydrodynamic size for fdIONP (*N* = 6); (**b**) Transmission electron microscopy (TEM) image of fdIONP; (**c**) Fourier transform infrared spectroscopy (FTIR) spectrum for fdIONP; (**d**) Field dependent magnetization of fdIONP and (**e**) Relaxivities (*r*_1_ and *r*_2_) measurements for fdIONP in water at 37 °C and 1.5 T.

This approach yielded extremely small nanoparticles with a hydrodynamic size, as determined by DLS, of 21.5 nm (PDI 0.18), and with an excellent reproducibility for all the repetitions of the procedure (Figure 1a). The surface charge was observed in a negative value, as expected, of −15.8 mV. Core size was also checked by transmission electron microscopy (TEM, Figure 1b and Figure S1) showing, as expected, a really small size of 2.5 ± 0.2 nm and a crystal size of 2.6 nm, according to X-ray powder diffraction (XRD) measurements (Figure S2). At higher magnification TEM images show the lattice planes of individual particles, indicating the individual particles to be monocrystalline (Figure S1). The difference between the core size and the hydrodynamic size is explained by the large polymeric layer surrounding the nanoparticle, as the thermogravimetric data demonstrates (Figure S3) with a weight loss of 80% at 300 °C, ensuring the colloidal stability of the nanoparticles. Characterization of surface composition by Fourier transform infrared spectroscopy (FTIR) of both FITC-dextran and the fdIONP showed the expected band for a successful synthesis, particularly at 1000 cm^−1^ and 1325 cm^−1^, and for iron oxide at 400 cm^−1^ and 545 cm^−1^, indicating the presence of dextran on the surface and Maghemite in the nanoparticles (Figure 1c). Magnetic properties of fdIONP were analyzed with a superconducting quantum interference device (SQUID), revealing a magnetic moment (Ms) of 17.2 emu/g Fe_3_O_4_ (Figure 1d). This small value was expected due to the really small size of the core and the consequent spin canting effect. This effect is due to the lack of full alignment of the spins in the surface. According to literature, the thickness of the spin-canted layer is about 0.9 nm and therefore our particles, with a hydrodynamic diameter of 2.5 nm, have 97.8% of the spins canted; higher than the previously reported extremely-small iron oxide nanoparticles [12,22]. Furthermore, a small saturation magnetization value was required to fulfill the goal of a positive contrast agent. Contrast agents’ capacity of reducing the relaxation times of the tissue in MRI is characterized based on the relaxivity values.

The relaxivity values for fdIONP were measured at 1.5 T and 37 °C showing a large value for *r*_1_ of 5.97 mM^−1^s^−1^ and a low *r*_2_ value of 27.95 mM^−1^s^−1^ (Figure 1e), giving *r*_2_/*r*_1_ ratio of 4.7. To be suitable as a T_1_ contrast agent for MRI, a compound must possess a high longitudinal relaxivity (*r*_1_) and the lowest possible *r*_2_/*r*_1_ ratio [12]. The results with our nanoparticles therefore predicted a good positive contrast. The high *r*_1_ value of fdIONP is due to the small size of the magnetic core, leaving a large number of Fe^3+^ ions, each with five unpaired electrons, on the surface of the nanoparticle.

### 2.2. Cell Labeling Studies

**Figure 2:**
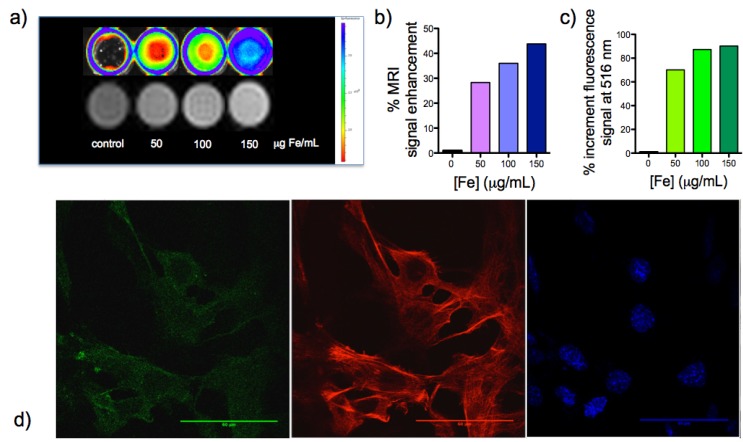
(**a**) Fluorescent imaging and magnetic resonance imaging (MRI) of labeled MAFs cells with fdIONP; (**b**) Percentage of signal enhancement in magnetic resonance images of labeled MAFs cells; (**c**) Percentage of signal enhancement in fluorescence images of labeled MAFs cells; and (**d**) Fluorescent confocal images of fdIONP-labeled cells at 80 µg/mL Fe concentration after 24 h of incubation, signal from fdIONP (green), phalloidin dye (red), and DAPI (blue).

To demonstrate the feasibility of generating positive contrast in MRI and the fluorescence from the surfactant we prepared phantoms with Mouse Adult Fibroblasts (MAFs) from C57BL/6 mice, incubated with increasing concentration of iron (0, 40, 80, and 120 μg/mL). MAFs were studied under MRI for T_1_-based bright contrast and fluorescence. MRI for the cells with highest Fe concentration showed the highest intensity of the bright contrast, while the intensity decreased and the image darkened with the decreasing Fe concentration (Figure 2a). This proved our particles to be a positive contrast agent in MRI very well. An epifluorescent image was collected and, as expected, the fluorescence intensity increased relatively with the increase in iron concentration in each of the sample. This was further confirmed by recording the relative fluorescence unit (RFU) of the samples at a wavelength of 516 nm in NanoDrop 3300. There was observed to be a linear correlation between the iron concentration in each of the samples and the hence-observed fluorescence intensity. The RFU values of the samples with 0 μg/mL (control), 40 μg/mL, 80 μg/mL, and 120 μg/mL were measured as 217.3, 726.5, 1705.4, and 2228.8, respectively. The percentage of signal enhancement by both imaging techniques can be calculated (Figure 2b,c) showing a quite good value of 30%–40% signal enhancement for MRI and about 65%–95% for fluorescence imaging, demonstrating a good labeling of the cells. This labeling was further confirmed by confocal images of the MAFs using DAPI staining for the nucleus, phalloidin568 for cytoskeleton and the fdIONP. As Figure 2d shows, there is a clear staining of the cells by the nanoparticles, very well correlating with the other dyes used (more images in Figures S4 and S5) and quantified by cytometry (Figure S6) where a clear increase is observed, while even considering some autofluorescence from the cells. Although there is an increase in fdIONPs uptake measured by flow cytometry in FITC laser, there is no a proportional increase in side scatter (SSC) microscopy, SSC parameters after 24 h of incubation with different concentrations of fdIONPs. SSC is related with cell complexity and nanoparticle-cell internal interaction. After analyzing flow cytometry results, confocal microscopy images in various depths within the sample (z-stacks), and low cytotoxicity in cells even with the highest Fe concentration, it can be concluded that most of the fdIONPs could be localized in the extracellular cell membrane and/or be surrounding it. Finally, proliferation studies demonstrated no cytotoxic effect at the concentration of fdIONP used, as expected, due to the composition of the particles (Figure S6).

### 2.3. In Vivo Magnetic Resonance Angiography

**Figure 3:**
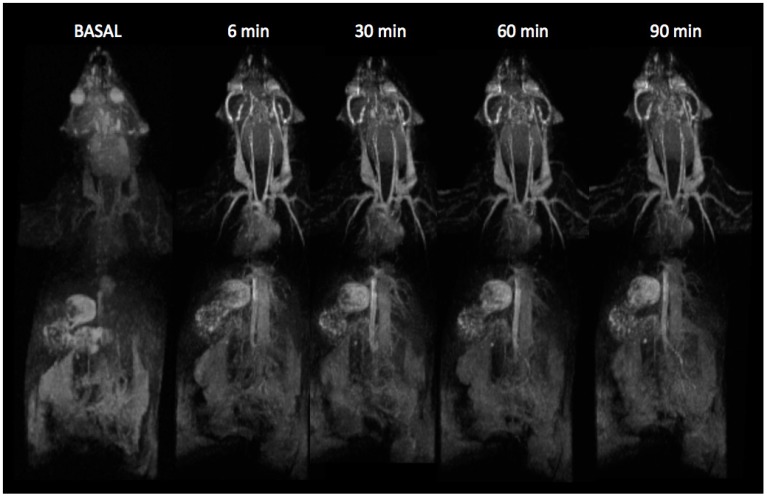
Magnetic resonance angiography of a mouse at increasing times after intravenous injection of fdIONP.

The utility of fdIONP for *in vivo* positive contrast MRI was investigated in mice, by injecting the nanoparticles at a dose of 2.2 mg Fe/kg into healthy animals. The short T_1_ relaxation time of these nanoparticles produces high signal intensity and excellent anatomical detail in Magnetic Resonance Angiography (MRA) acquisitions. The views generated, clearly depict the main vascular architecture, carotids, subclavian, abdominal aorta and heart chambers, and some smaller vessels (Figure 3). The high quality of small-vessel imaging was maintained even 90 min post injection (Figure 3) due to the small size and thick polymeric layer which was possible to obtain through a one-step protocol in the microwave. This highlights an important advantage of these nanoparticles. Due to their small size and biocompatible surface coating, they remain in circulation much longer than most nanoparticles, providing, hence, an excellent contrast for blood pool applications and a platform for functionalization and targeted molecular imaging.

## 3. Experimental Section

### 3.1. Preparation and Characterization of fdIONP

FITC CM Dextran (4 kDa) coated iron oxide nanoparticles were synthesized in a CEM microwave unit. All the necessary chemicals involved in the experiment, were purchased from Sigma-Aldrich Quimica SL(Madrid, Spain), and used without any further purification, while distilled water was used as supplied from our institution (Centro Nacional de Investigaciones Cardiovasculares, Madrid, Spain). Briefly, in an ideal 5 mL reaction, 37.5 mg (0.1387 mmol) of iron (III) chloride hexahydrate; FeCl_3_·6H_2_O was taken as the iron precursor in a 5 mL microwave adaptable tube. To this was added, 100 mg (25 mmol) FITC-CM dextran and was together formed into a homogeneous solution, with 4.5 mL of distilled water. The magnetic stirring bead was dropped in the tube and, finally, 0.5 mL of hydrazine hydrate was added before placing closed container inside the microwave unit. The reaction was carried out at a temperature of 100 °C and with the irradiating power set at 240 W. The reaction continued under high stirring for 10 min. The sample, thereafter, as cooled down to 60 °C in 2 min through the self-equipped cooling mechanism of the instrument, before being extracted out. The preliminary sample purification step, involved filtration through a PD10 desalting columns (GE Healthcare, Madrid, Spain) eluted first with 15 mL of distilled water. In the first step, 2.5 mL of the prepared sample was subjected to the column for purification. The sample retained from this step had an increase in dilution with a final volume of 3 mL. This 3 mL of the elute was further purified through Amicon Ultra 0.5 mL Centrifugal Filters (Merck Millipore, Madrid, Spain) with a cut-off of 30 kDa. The recovered sample was, thereafter, made up to its initial volume of 3 mL and used as such for further characterizations and studies. The hydrodynamic size, surface charge and the polydisperstity index (PDI) of the obtained nanoparticles (fdIONP) was determined through Zetasizer Nano ZS (Malvern Instruments, Worcester, UK), equipped with 633 nm He-Ne laser. The Fe quantification in the sample was done through ICP. Size measurements were conducted on a JEOL 3000 F transmission electron microscope, with an accelerating voltage of 300 kV, samples were deposited in a Cu grid dispersed in water.

### 3.2. XRD

The crystal structure of the samples was identified by X-ray powder diffraction in a Bruker D8 Advance powder diffractometer (Bruker Spain S.A. (BBIO & BOPT) S.A. Madrid, Spain), using Cu Kα radiation with an energy-discriminator (Sol-X) detector. Patterns were registered within 10 and 80 in 2θ at 0.01 degrees per second. The average crystallite size was calculated with Scherrer’s equation from the half-width of the (311) diffraction peak. The XRD spectra correspond to an inverse spinel structure. The error in the crystallite sizes is 0.1 nm, and is mainly due to the instrumental line width (Δ2θ = 0.11).

### 3.3. Relaxivity Studies

T_1_ and the T_2_ relaxation times were studied in 1.5 T Bruker Minispec TD-NMR mq60 (Bruker Spain S.A. (BBIO & BOPT) S.A. Madrid, Spain) at a gain of 51 dB. The *r*_1_ and *r*_2_ values in each respective case, were defined by the slope plotted between the iron concentration, and the R_1_(1/T_1_)-R1_b_(1/T_1blank_) and R_2_(1/T_2_)-R_2b_(1/T_2blank_). The sample was studied under four dilutions of 92.5%, 85%, 77.5%, and 75%.

### 3.4. Cell Labeling Studies

#### 3.4.1. Cell Culture and Media

C57BL/6 mouse adult fibroblasts (MAFs) were grown in DMEM (Dulbecco’s Modified Eagle Medium) supplemented with 5% fetal bovine serum (FBS), 1% penicillin-streptomycin and 1 mM sodium pyruvate in a humidified atmosphere of 5% CO_2_ at 37 °C. Cytotoxicity and nanoparticle uptake were assessed in MAFs exposed to fdIONP at different concentrations. Control cells were treated with vehicle.

#### 3.4.2. fdIONPs Uptake and Cytotoxicity Assays

After culturing for 24 h with fdIONP (40, 80 and 120 µg/mL Fe concentrations), cells were trypsinized and measured in PBS. DAPI, the cell viability marker, was then added at a final concentration of 0.001% (*w*/*v*). A total of 10,000 events were recorded for each simple using the BD FACSCanto™ II system BD Biosciences, Madrid, Spain. All experiments were performed in triplicate. Samples were analyzed with BD FACSDiva™ Software (BD Biosciences, Madrid, Spain) and FlowJo Software (version 10.0.7.12, FlowJo LLC, Ashland, OR, USA).

#### 3.4.3. *In vitro* Inmunofluroescence Assay

Cells seeded on coverslips, were washed in PBS, fixed (4% formaldehyde) and permeabilized in PBS with 0.1% Triton X-100. Samples were incubated with Alexa Fluor^®^ 568 Phalloidin (1:50, Life Technologies, Carlsbad, CA, USA) for 45 min in blocking solution (5% BSA). Phalloidin dye is used to visualize F-actin in cytoplasm of cultured cells. Slides were washed twice with PBS and distilled H_2_O and applied with, ProLong^®^ Gold Antifade Reagent with DAPI to stain cell nucleus. Optical sections were acquired using a Leica TCS SP5 confocal system and LAS AF 2.6.0 software (Leica Microsystems, Barcelona, Spain).

### 3.5. MRI

Cells were labeled with different fdIONP dilutions (different Fe conc.) for 24 h. After fdIONP incubation, cells were trypsinized, washed three times with PBS and collected in tubes. Samples were first studied in phantoms for T_1_ mapping in MRI. For *in vivo* MRA, mice weighing 20 g were anesthetized with 2% isoflurane and oxygen before being placed on a thermoregulated (38 °C) mouse bed. Ophthalmic gel was added in their eyes so as to prevent retinal damage due to drying. The MRI equipment used in this study was an Agilent/Varian scanner (Agilent, Santa Clara, CA, USA) equipped with a DD2 console and an active shielded 205/120 gradient insert coil with 130 mT/m maximum gradient strength and a combination of volume coil/two channel phased-array (Rapid Biomedical GmbH, Rimpar, Germany). 3D gradient echo with magnetization transfer contrast (MTC) prepulse MRA was performed with the following parameters: min TR, 12.64 ms; min TE, 2.32 ms, flip angle 20, 2 averages, acquisition matrix 192 × 128, MTC flip angle 810 deg; duration, 6 ms; offset frequency, 2000 Hz.

### 3.6. Fluorescence Studies

The fdIONP-labeled cells were expected to demonstrate fluorescence due to the FITC labeling of the surface coating dextran, in this case. No secondary fluorescence labeling was done for any further studies. The cells incubated with different iron concentrations (different fdIONP dilutions), same as the ones used for phantom studies in MRI, were studied for fluorescence in comparison with the un-doped cells as the negative control. The epifluorescence study was performed in a pre-clinical In Vivo Imaging System (IVIS) from PerkinElmer Inc. (Waltham, MA, USA). The relative fluorescence was also observed through NanoDrop 3300 Fluorospectrometer (Thermo Scientific, Waltham, MA, USA) at a wavelength of 516 nm, specific for FITC CM Dextran.

## 4. Conclusions

We here demonstrate how the use of microwave synthesis enables an extremely fast and robust production of fluorescent nanoparticles, with an excellent performance as a positive contrast for MRI. Moreover, we get these results in an easy and reproducible, one step protocol from the precursors. Furthermore, the presence of a large polymeric coating and reduced size has a dramatic enhancement of the circulating time of the nanoparticles in blood. This same thick surfactant will enable further functionalization for targeted molecular imaging.

The ease of the synthesis and purification protocols renders high-quality nanoparticles that can be used for fluorescence imaging, *in vitro* cell imaging, and *in vivo* imaging for MRA. This is the first time, to our knowledge, that T_1_-MRI nanoparticles with fluorescent signal are produced by microwave technology.

## Supplementary Materials

Supplementary materials can be accessed at: http://www.mdpi.com/2079-4991/5/4/1880/s1.
