# Supplementary material for: T1-MRI Fluorescent Iron Oxide Nanoparticles by Microwave Assisted Synthesis
Source: Nanomaterials (Basel). 2015 Nov 4;5(4):1880–90. doi: 10.3390/nano5041880 (PMC5304808; doi:10.3390/nano5041880)
Supplement: Supplementary file 1 [file nanomaterials-05-01880-s001.pdf]

Supplementary Information

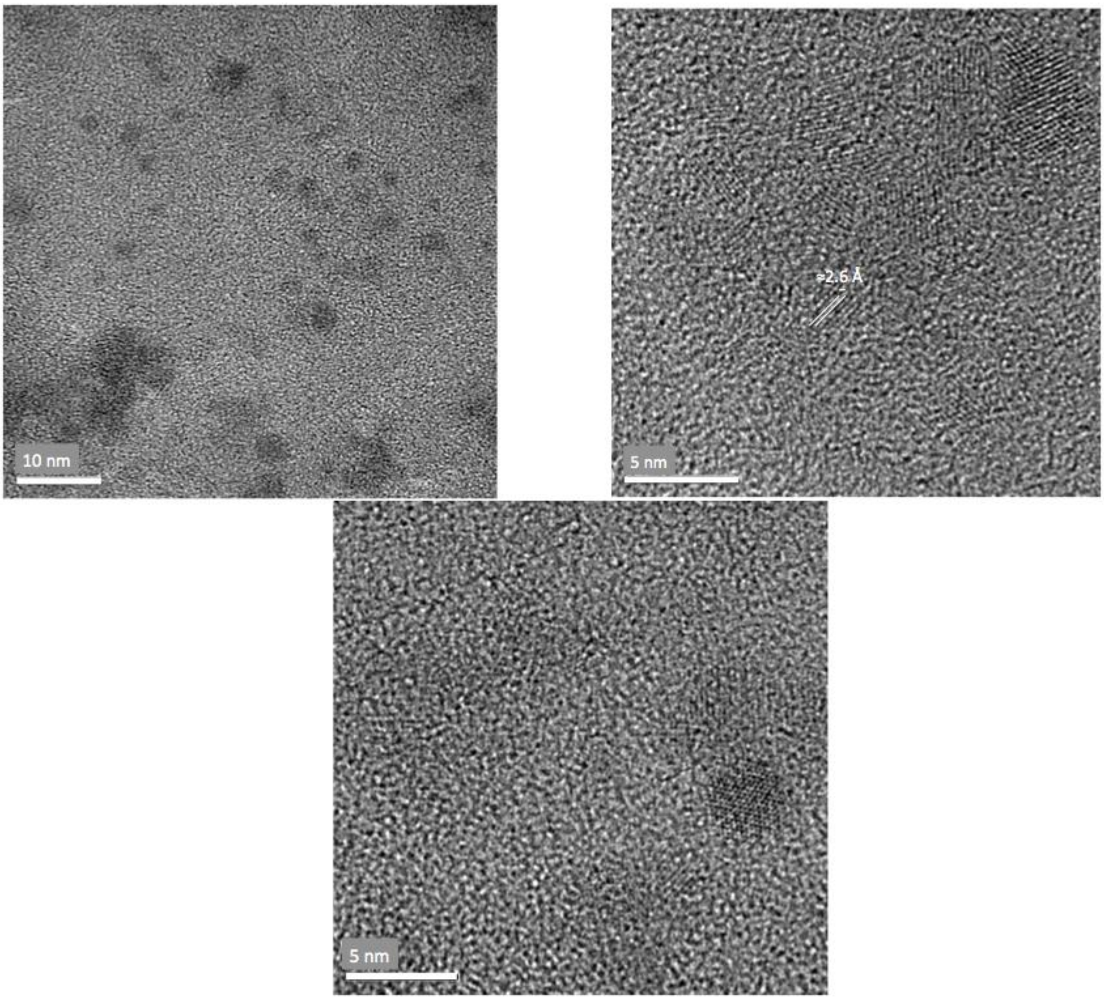

Figure S1. TEM images of fdIONP at different magnifications.

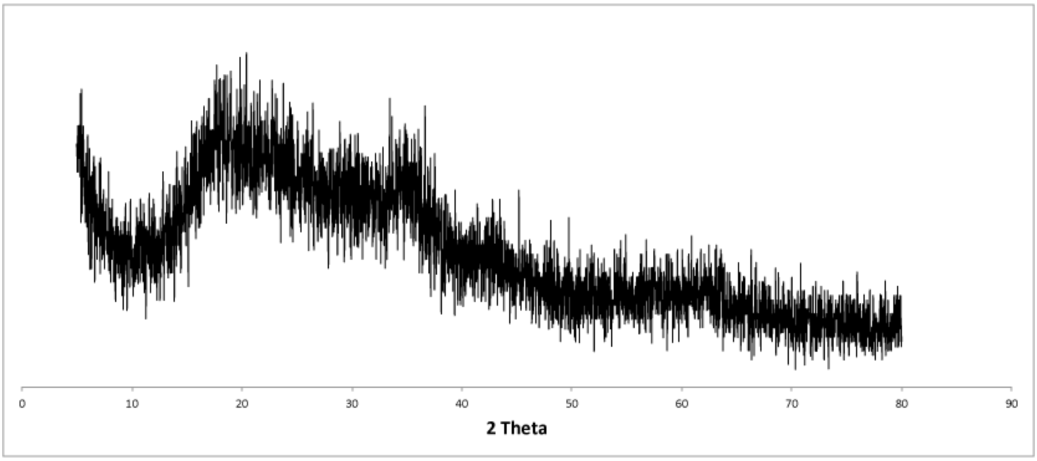

Figure S2. XRD plot for fdIONP.

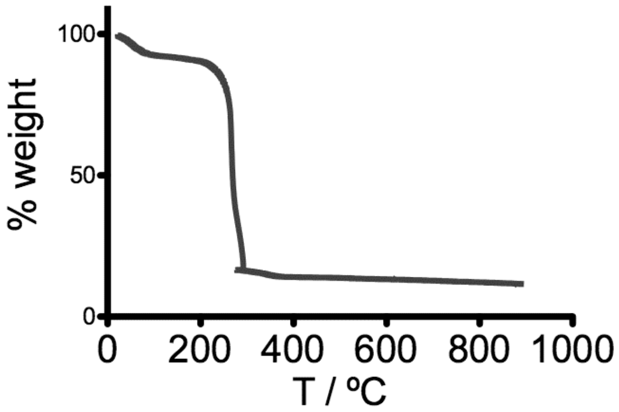

Figure S3. Thermogravimetric curve for fdIONP.

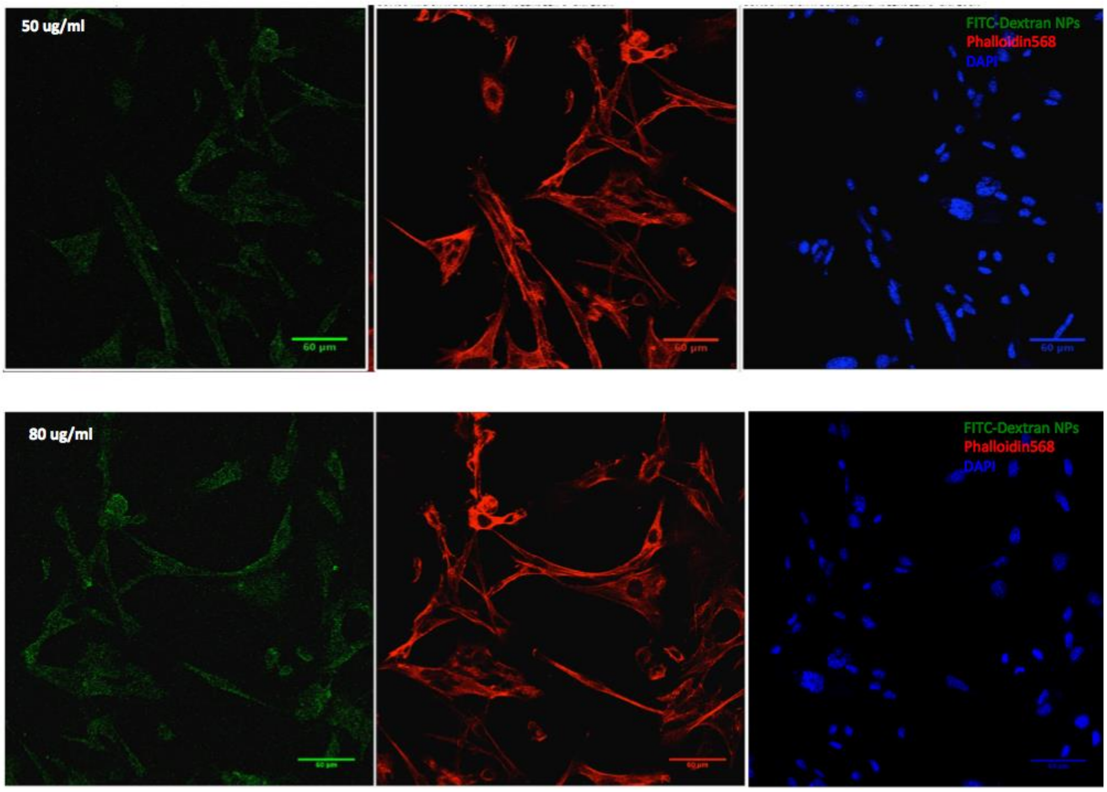

Figure S4. Fluorescent confocal images of fdIONP labelled cells at 50 µg/mL Fe and 80 µg/mL concentration after 24 h of incubation, signal from fdIONP (**green**), phalloidin dye (**red**) and DAPI (**blue**).

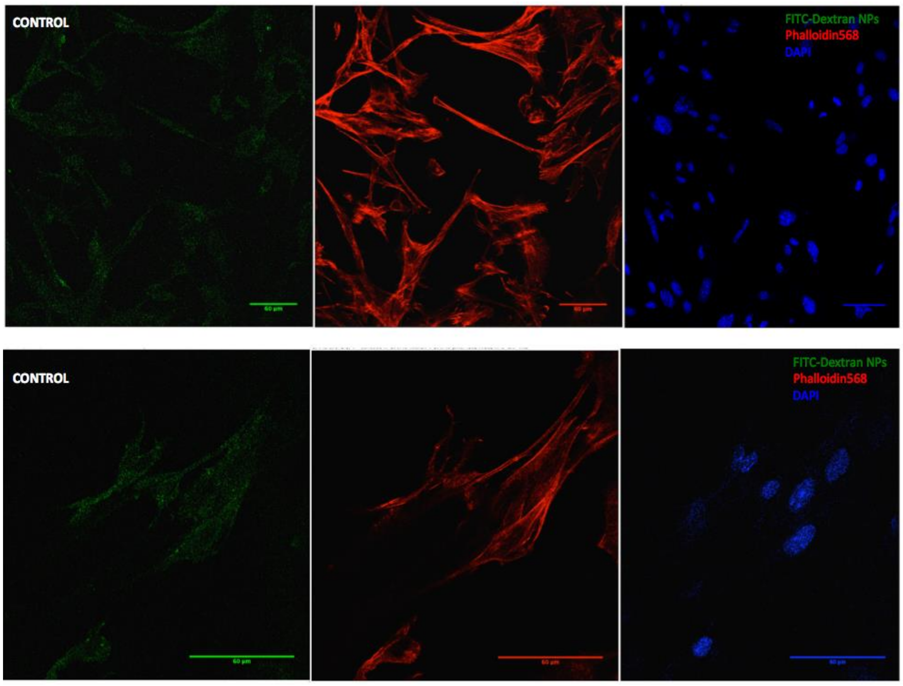

Figure S5. Fluorescent confocal images of fdIONP control cells after 24 h of incubation, signal from fdIONP (**green**), phalloidin dye (**red**) and DAPI (**blue**).

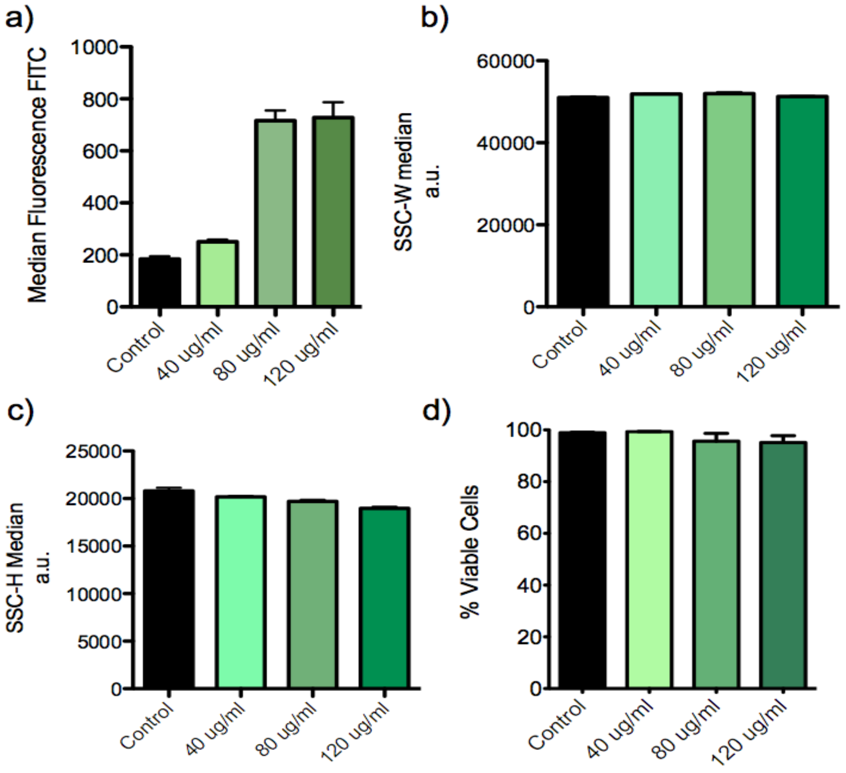

Figure S6. Values for the labelled cells with different concentrations of fdIONP for (**a**) Median fluorescence; (**b**) SSC-W; (**c**) SSC-H and (**d**) percentage of viable cells.

© 2015 by the authors; licensee MDPI, Basel, Switzerland. This article is an open access article distributed under the terms and conditions of the Creative Commons Attribution license (http://creativecommons.org/licenses/by/4.0/).
